# Connectivity between Migrating and Landlocked Populations of a Diadromous Fish Species Investigated Using Otolith Microchemistry

**DOI:** 10.1371/journal.pone.0069796

**Published:** 2013-07-29

**Authors:** Ingrid Tulp, Marieke Keller, Jacques Navez, Hendrik V. Winter, Martin de Graaf, Willy Baeyens

**Affiliations:** 1 Institute for Marine Resources and Ecosystem Studies (IMARES), Wageningen University, IJmuiden, The Netherlands; 2 Department of Analytical and Environmental Chemistry, Vrije Universiteit Brussel, Brussels, Belgium; 3 Section de Pétrographie-Minéralogie-Géochimie, Koninklijk Museum voor Midden-Afrika, Musée Royal de l'Afrique Centrale, Tervuren, Belgium; SUNY College of Environmental Science and Forestry, United States of America

## Abstract

Smelt *Osmerus eperlanus* has two different life history strategies in the Netherlands. The migrating population inhabits the Wadden Sea and spawns in freshwater areas. After the closure of the Afsluitdijk in 1932, part of the smelt population became landlocked. The fresh water smelt population has been in severe decline since 1990, and has strongly negatively impacted the numbers of piscivorous water birds relying on smelt as their main prey. The lakes that were formed after the dike closure, IJsselmeer and Markermeer have been assigned as Natura 2000 sites, based on their importance for (among others) piscivorous water birds. Because of the declining fresh water smelt population, the question arose whether this population is still supported by the diadromous population. Opportunities for exchange between fresh water and the sea are however limited to discharge sluices. The relationship between the diadromous and landlocked smelt population was analysed by means of otolith microchemistry. Our interpretation of otolith strontium (^88^Sr) patterns from smelt specimens collected in the fresh water area of Lake IJsselmeer and Markermeer, compared to those collected in the nearby marine environment, is that there is currently no evidence for a substantial contribution from the diadromous population to the spawning stock of the landlocked population.

## Introduction

Smelt *Osmerus eperlanus* occurs along the coast of Europe from North West Russia in the north, including the White Sea and Baltic Sea, to the Bay of Biscay in the south [1]. Anadromous smelt resides in estuaries originally, but several populations have become landlocked due to geological processes or habitat alterations by man [2]. Many fish species are known for their large phenotypic plasticity in life history traits in relation to environmental characteristics. Similarly smelt has shown the ability to adapt to new circumstances in many locations. Smelt can show diverse life history traits among populations, ranging from migratory (diadromous) to resident in freshwater [3].

**Figure 1:**
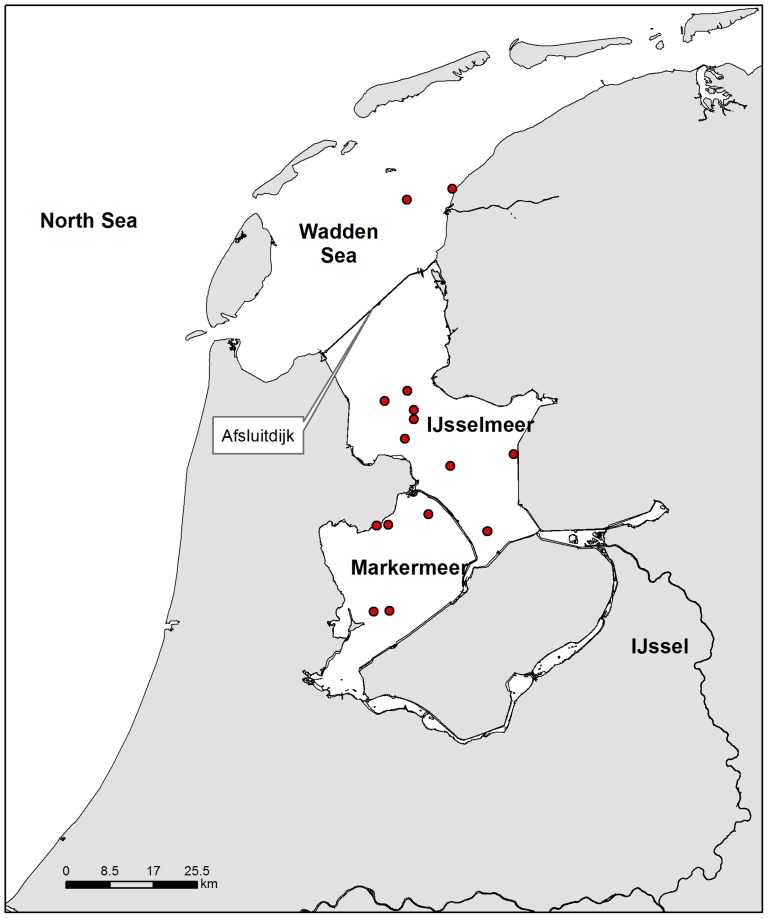
Map of the study area showing the locations where samples were taken.

Smelt was abundant in the former Zuiderzee estuary (The Netherlands) before its closure with a large dam (Afsluitdijk) in 1932 (Fig. 1). After the Afsluitdijk was built, the Zuiderzee estuary was closed off from the sea resulting in the freshwater Lake IJsselmeer, in which a landlocked population of smelt developed. Migrating opportunities became severely reduced to instants when dam sluices are opened and water current is low enough to allow passage. Diadromous smelt still inhabit coastal waters and the Wadden Sea and can grow up to 25 cm and eight years old, and become mature at 3–4 years [2]. The landlocked smelt morphs become mature after only one year and stay smaller (10–12 cm) than anadromous smelt morphs [4]. Smelt spawn in early spring in freshwater, preferably in running water in tributaries of lakes or along shallow shores of lakes and rivers, on firm surfaces such as sand, gravel and stones [3]. The majority of landlocked smelt spawn only once and individuals bigger than 12 cm (and presumably older than 2 years) make up around 1% of the entire landlocked population (Drost & de Witte unpubl.).

The smelt populations of IJsselmeer and Markermeer have been declining strongly since 1990 [5]. In winter, the Lake IJsselmeer area is home to the largest population of waterfowl in Western Europe [6]. Based on the number of (among other groups) fish eating birds both lakes are protected as wetlands of international importance for water birds in the Ramsar Convention and have been assigned as Natura 2000 areas. Smelt fisheries used to be an important activity locally and are carried out during the spawning season, from mid-February till mid-March, when large numbers aggregate near hard substrate areas, such as dykes [7]. Because of low biomass, smelt fishery was closed during five years in the last decade (unpubl. data).

The life history of anadromous fish depends on both the quality of their habitat and the connectivity between habitats After the creation of both lakes two possible developments may have occurred: 1) smelt became separated in a landlocked and a diadromous population, with the landlocked population closing its life cycle within the lakes and the diadromous population using spawning sites elsewhere in areas adjoining the Wadden Sea; 2) the two populations are mixed and the diadromous population consists of the individuals washed out through the sluices; returning from the Wadden Sea to Lake IJsselmeer is limited to events when sluices in the dyke are opened.

Given the decline in the landlocked smelt and the potential for recovery of the population, the question we aim to answer is: do anadromous smelt still contribute to the total spawning biomass of smelt in Lake IJsselmeer and Markermeer? Could the landlocked smelt stock be resupplied with Wadden Sea smelt? Insight in the importance of the marine stock for the landlocked stock would contribute to the understanding of the population dynamics of smelt, and consequently of those bird species that rely on smelt as a food source.

A method commonly used to distinguish freshwater from marine origin in fish is comparing the Strontium (^88^Sr) profiles along sagittal otoliths [8], [9], [10], [11], [12]. The utility of this method stems from the fact that otoliths are largely metabolically inert, deposit a thin layer calcium carbonate continuously with age and reflect ambient chemistry [13]. The origin of smelt collected at different locations in Lake IJsselmeer and Markermeer during the peak spawning period was investigated, by comparing the Strontium (^88^Sr) signature of their sagittal otoliths with those of specimens collected in the Dutch Wadden Sea (Fig. 1). If the diadromous population still contributes to the landlocked population, we expected to find individuals with a marine signal in their ^88^Sr otolith profiles in the spawning period. The ^88^Sr concentrations in the edge of the otoliths (the part most recently deposited) of Wadden Sea individuals was used as a marine reference. These concentrations were compared to ^88^Sr concentrations in specimens collected in both lakes at spawning time.

## Methods

### Study Area

The estuary Zuiderzee was closed off from the Dutch Wadden Sea sea in 1932 by a dam resulting in the formation of the freshwater lake IJsselmeer (Fig. 1). This was part of a larger plan with a threefold aim: protecting central Netherlands from the effects of the North Sea, increasing the Dutch food supply with new agricultural land and improving water management by creating a lake from the former uncontrolled salt water inlet. This lake is nowadays the largest freshwater body in the Netherlands. It is a shallow lake with a mean depth of 4 m and with up to 8 m deep channels. The lake was further divided by three land reclamation projects, reducing its surface with 40% to about 1900 km^2^ [5]. The lake Markermeer with a surface area of 700 km^2^, was created after the construction of a dam in 1975, with the objective of reclaiming Lake Markermeer, a plan that was later abandoned.

Lake IJsselmeer gets nutrient input from the River IJssel, a tributary of the River Rhine. Primary production and fish production in Lake Markermeer is lower than in Lake IJsselmeer [14], [15]. Both lakes have multiple functions, fisheries, recreation, drinking water supply, transport and as a rest and forage area for birds [16]. Nutrient reductions in the early 1980s lead to a drop in phosphate levels. In Lake IJsselmeer retention time of water is around 6 months, whereas in Lake Markermeer it is around 12 months [5].

Migratory possibilities for smelt to pass the different dams are limited. In the Afsluitdijk, two sluice groups (one in the West and one in the East) control the outflow of fresh water and there are two shipping locks. These are the only direct connections between lake IJsselmeer with the IJssel and Rhine river basin. The westerly sluice complex has three groups of discharge sluices with five tubes in each group. The easterly sluice complex has two groups with five tubes each. Each tube is 12 m wide. Instead of the former opening of 35 km, nowadays only 25 sluices measuring 12 m form the connection between the Wadden Sea to IJsselmeer, a reduction of 99%. The discharge is governed by rain fall, so in spring discharge is generally high and chances for inland migration, especially for weak swimmers, are limited to periods at the end of discharge periods when the water level outside and inside the dike are at a similar level. Migration possibilities have not changed greatly since the closure in 1932. The nearest possibility for smelt to enter an unobstructed freshwater area and spawn is the Ems-Dollard estuary, which is ca 150 km East from the Afsluitdijk. Migration possibilities between Lake IJsselmeer to Lake Markermeer and reverse are limited to two complexes, at the North side there are two shipping locks and a discharge sluice (two tubes of 20 m wide) and at the South side there is a shipping lock and a discharge sluice (six tubes of 16 m wide).

**Figure 2:**
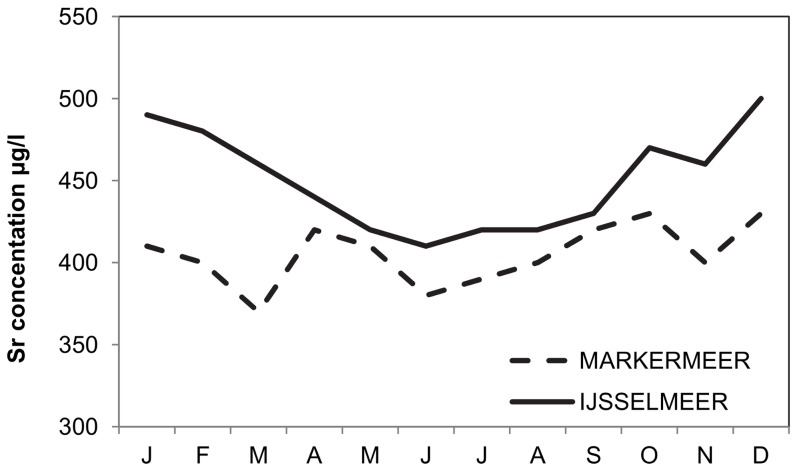
Seasonal variation of Strontium concentrations in Markermeer and IJsselmeer in 2009 (data Rijkswaterstaat).

Mean monthly background ^88^Sr values vary between IJsselmeer and Markermeer, with lower levels during summer than winter and a generally lower level in Markermeer than in IJsselmeer (Fig. 2, data standard monitoring Rijkswaterstaat, concentration measured in surface water). Similar data for the Wadden Sea are not available.

### Sample Collection

In March 2010 smelt were sampled with a 2,5 m beam trawl (rigged with fine-meshed cod-end; 0.5 cm) in Lake IJsselmeer/Markermeer. All known spawning aggregations were sampled (Fig. 1). Adult fish were collected from the Wadden Sea with a 3 m beam trawl (rigged with a fine-meshed cod-end; 20 mm) between April and October 2009 to serve as a marine reference. After capture fish were kept frozen and taken to the laboratory for later dissection. All fish were measured to the nearest mm. In total 110 smelt, 56 from IJsselmeer (collected in 4 hauls/locations) and 54 from Markermeer (4 hauls/locations, length 5–7 cm, two specimens of 11 and 16 cm), and 9 smelt (length 13–23 cm) from the Wadden Sea (2 hauls/locations) were collected. The permission to collect fish in the areas under study was issued by the Ministry of Economic Affairs in the Netherlands, department implementation fisheries regulation (nr 60898). In The Netherlands there is no need for special approval to catch or kill fish by an ethics committee (the experiments on animals act, BWBR0003081). Smelt is a fragile fish and most of them were already dead when taken on board. For the few fish still alive, suffering was ameliorated by immediate freezing.

The total length of all specimens were measured (±1 mm) and otoliths were collected in the laboratory. One otolith from each specimen was cleaned and embedded in resin. The otoliths were placed on a first layer of nearly cured resin, after which the otoliths were fixed evenly with a second layer of resin. Embedded otoliths were sectioned through the nucleus. The thickness of the slides ranged between 0.5 and 0.6 mm. Three resin strips, containing 10 sectioned otoliths each, were glued to a glass slide with clear resin. No coverslip was used. The slides were stored in covered containers until analysed by laser ablation (LA) inductively coupled mass spectrometry (ICPMS).

### Laser Ablation-ICP-MS Analyses

An ICP-MS (Thermo Scientific X-serie2) coupled to a laser (ESI New Wave UP-193 Fast Excimer ArF) was used to determine the concentration of ^88^Sr along sagittal otoliths. The NIST612 reference material was used for the optimization of following instrumental parameters: the movement rate, the dwell time, the flow of Ar, the flow of He, the output and the repetition rate. The choices were made according to the intensities and stabilities measured. Logically, with a longer dwell time, the stability increases. The configuration set allowed a resolution of 3.7 µm, an ablation diameter of 25 µm (35 µm at the pre-ablation point) and a scan speed of 25 µms^−1^).

In order to better answer our questions, a quantitative analytical method was developed instead of working with ^88^Sr/^44^Ca ratios. To do this, calibration standards NIST 612 and 610 (lines of 600 µm) were chosen. Finally, to correct the drift and differences in efficiency, the ^43^Ca was used as the internal standard. Given the size of smelt otoliths (ca 1 mm), the analysis is fast enough and the extent of standards was estimated every 10 samples. To calculate concentrations, normally the standards enclosing the samples were chosen. Given the stability of the measures (r^2^ = 0.99), all the concentrations were calculated based on a line with all standards measured during one day. In preliminary tests, the resin itself was measured and it showed a very special signature, completely different from the otoliths. Such signatures were not observed in the otolith measurements. In addition, to avoid the risk of contamination by the resin or by dust, the line of measurement was situated in the middle of the sample and avoided the extreme edges. Moreover a pre-ablation was carried out. Tests indicated that a third ablation did not differ from the second one. Hence, all results refer to the second ablation measurement that was always preceded by a pre-ablation step. All the measures are in TRA mode (time resolved analyses). The laser was led through the core of the otolith.

### Data Analyses

Of each profile cross-cutting the otolith from the rostrum, through the nucleus, to the post-rostrum, the part from the nucleus to the rostrum was used for analyses. ^88^Sr at the cores show the conditions the fish encountered lived when they began to grow. Only the outer 50 measurements (roughly representing 185 µm) of Wadden Sea individuals were used to define the threshold for the marine signal, to ascertain that the ^88^Sr concentration truly represented the marine environment and to avoid potential ontogenetic effects in ^88^Sr deposition. Similarly the last 50 measurements (outer edge) of the otoliths of fish caught in the lakes were chosen to represent the conditions at time and shortly before time of capture.

Specimens originated from different hauls, but a varying number of individuals were collected from each haul. Therefore, the overall differences between the concentrations of ^88^Sr concentration in the section near the edge of the otoliths from specimens collected in the Wadden Sea and both lakes were tested with a mixed effect model with individual nested within haul as random effect and location as fixed effect [17]. The random intercept allow for correlations between observations from the same site and individual. Because of heterogeneity in the data, ^88^Sr concentrations were log-transformed prior to analyses.

All data analyses were carried out in R 2.15.1.

## Results

**Figure 3:**
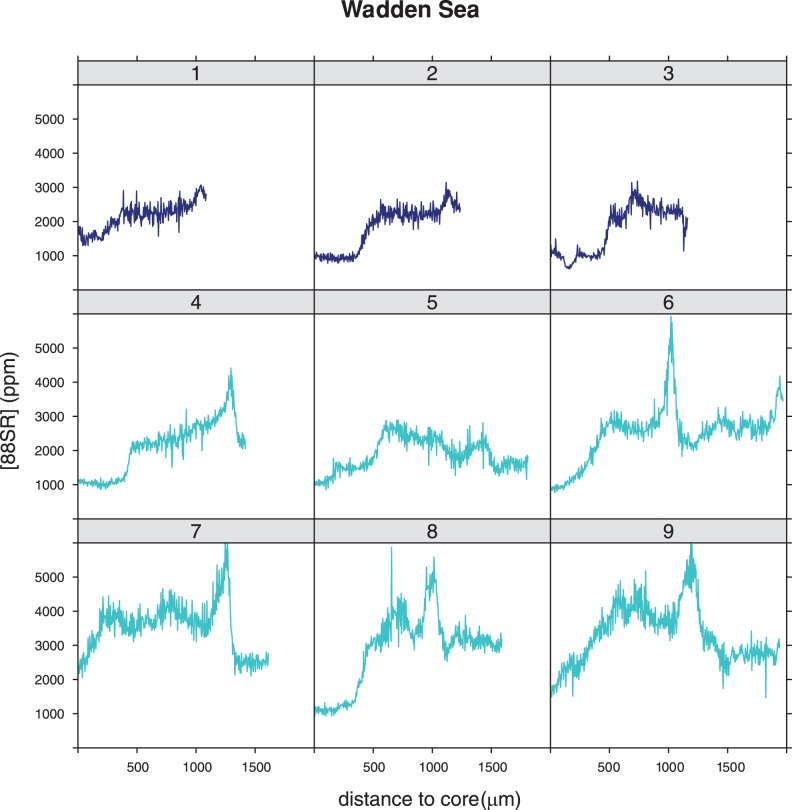
Strontium concentration profiles (in ppm) for smelt caught in the Wadden Sea, going from the nucleus (left) to the outer edge (right). The colours indicate the different hauls, all profiles in one colour originate from the same haul.

**Figure 4:**
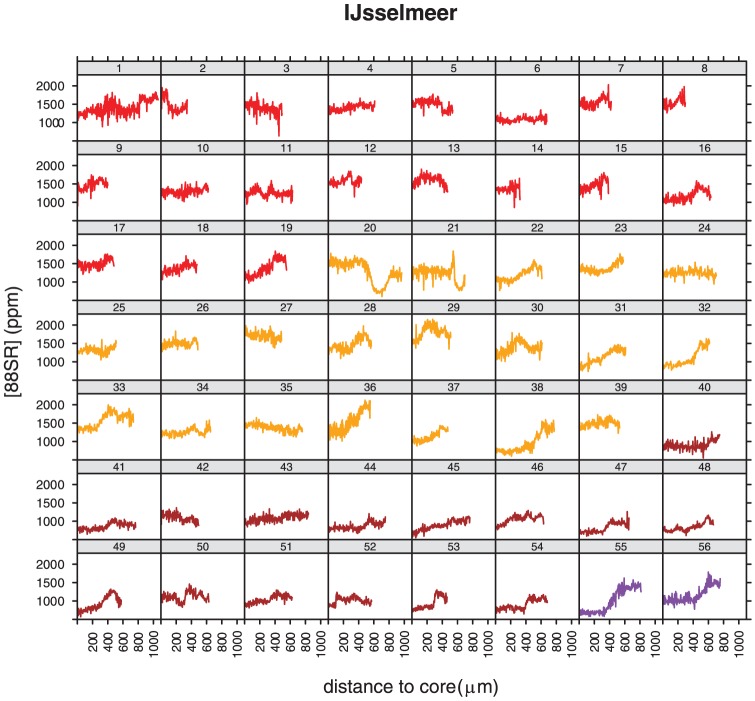
Strontium concentration profiles (in ppm) for smelt caught in Lake IJsselmeer, going from the nucleus (left) to the outer edge (right). The colours indicate the different hauls, all profiles in one colour originate from the same haul.

**Figure 5:**
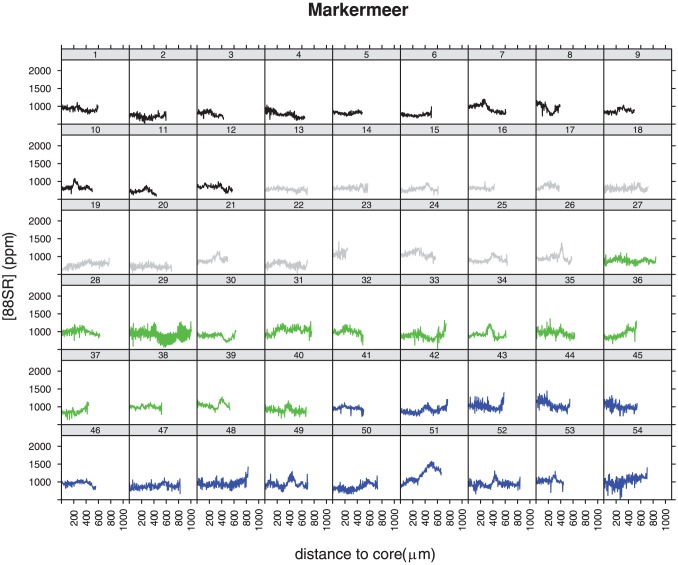
Strontium concentration profiles (in ppm) for smelt caught in Lake Markermeer, going from the nucleus (left) to the outer edge (right). The colours indicate the different hauls, all profiles in one colour originate from the same haul.

^88^Sr profiles of individuals caught in the Wadden Sea showed strong variations with ^88^Sr levels at the outer edge generally varying between 2000 and 6000 ppm, but not in the core (Fig. 3). The core values were all below 2000 ppm. The level at the outer edge, the part of the otolith that was most recently deposited, was used as a reference for the marine environment. Eight out of the nine individuals showed mean values greater than 2000 in the final 50 measurements (mean of the final 50 measurements of the 9: 2260 ppm (range 1600–3100 ppm). Individual 5 from the Wadden Sea has a different profile from the other eight, showing values below 2000 ppm at the edge. The overall pattern of this individual is however different from IJsselmeer individuals, that never show values>2000 ppm. The individual profiles of all specimens collected in fresh water showed ^88^Sr levels ranging between 500 and 2000 ppm. Profiles of specimens in Lake IJsselmeer were more variable than those from Lake Markermeer. In Lake IJsselmeer more than half of the individuals showed lowest values at the core and an increase in ^88^Sr concentration going from the core to the edge, but never as high as the level identified as marine (2260 ppm) (Fig. 4). Individuals from the 3^rd^ haul (brown, 40–54) had lower values at the core (500–1000) than most specimens from other hauls. Levels varied between 500–2000 ppm. The two largest animals (Fig. 4, individuals 20 and 21) had a distinct pattern: after constant flat profiles both showed a strong dip in ^88^Sr concentrations to values<1000 ppm. Profiles from Lake Markermeer specimen's otoliths were generally flat, with no clear trend going from the core to the edge and no obvious differences between individuals originating from different hauls (Fig. 5). ^88^Sr concentrations varied between 500–1200 ppm.

**Figure 6:**
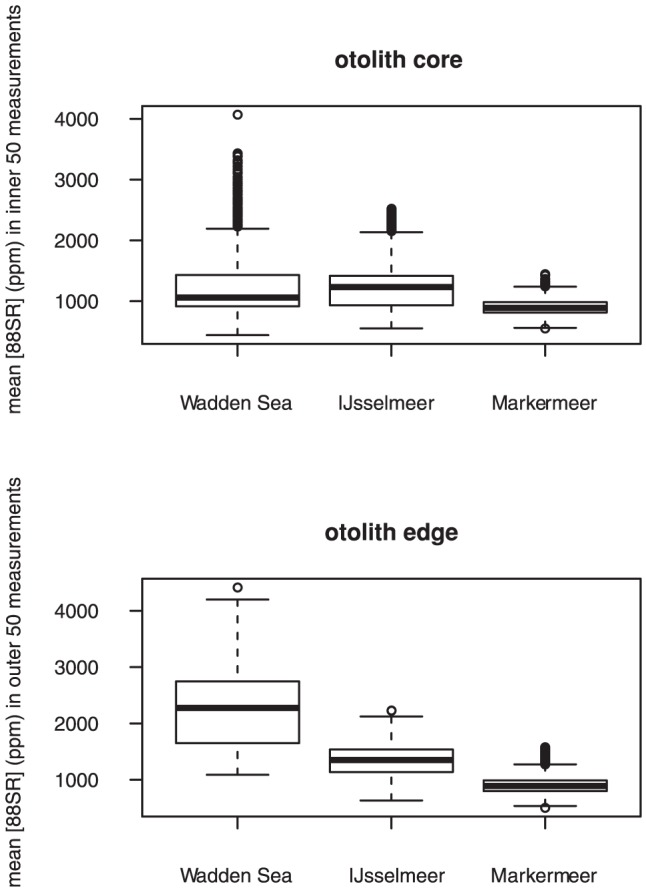
Mean Sr (in ppm) concentrations in otoliths of the inner 50 measurements (core) and the outer 50 measurements of smelt (edge) caught in the Wadden Sea, Lake IJsselmeer and Lake Markermeer.

Mean ^88^Sr concentrations at the otolith edge were significantly higher in the Wadden Sea than in both lakes, but also differed between the two lakes, with lowest values found in Lake Markermeer (Table 1, Fig. 6). At the core ^88^Srconcentrations did not differ significantly between any of the areas (Table 1, Fig. 6). Although mean core values were generally lower at lake Markermeer the difference was not statistically significant (Fig. 6).

**Table 1:** Estimates, estimated degrees of freedom and significance for parameters testing area effects for log^88^Sr concentrations at the core and the edge of the otoliths in mixed models with individual fish nested within sample as random effects.

response variable	parameter	estimate	standard error	df	t-value	P value
^88^Sr at edge	intercept	3.1207	0.03942	6142	79.164	0.0000
	area: Markermeer	−0.1715	0.05524	9	−3.105	0.0126
	area: Wadden Sea	0.1866	0.05688	9	3.281	0.0095
^88^Sr at core	intercept	3.0323	0.04071	6172	74.477	0.0000
	area: Markermeer	−0.0831	0.05672	9	−1.465	0.1767
	area: Wadden Sea	0.0171	0.05995	9	0.285	0.7815

## Discussion

### Connectivity and Dispersal between Freshwater and Marine Environment

The ^88^Sr concentrations in otoliths of individuals collected in the freshwater lakes were notas high as recorded in the otolith edges of specimens caught in the Wadden Sea. Therefore, based on our study we argue that there is no evidence of a substantial contribution from diadromous individuals to the landlocked smelt population. However, the possibility for a contribution can not be completely excluded. Observations near the discharge sluices,have shown that Wadden Sea smelt occasionally enters Lake IJsselmeer (Kruitwagen unpubl. data). Spawning generally takes place within a few weeks [18]. Due to ship time limitations the specimens were collected in a short period in March only, but covered all known spawning locations in Lake IJsselmeer/Markermeer. The sampling gear used is probably suboptimal for catching larger size classes of smelt if they had occurred. Large smelt were caught in fyke nets or gillnets in Lake IJsselmeer/Markermeer in the late 20^th^ century. Currently, these size classes are never encountered on the market or auction anymore (unpubl.data). A scenario in which the larger diadromous smelt enter Lake IJsselmeer and possibly even Markermeer for a very short period and in low numbers is possibly. Oriadromous smelt may quickly pass Lake IJsselmeer and head to historically known spawning sites, such as River IJssel [19]. In such scenario the recruits still contribute to the population in Lake IJsselmeer. Given the fact that older and larger individuals in most fish species (and also shown in smelt) contribute disproportionally to the recruitment [20], [21], a contribution from the diadromous to the landlocked morphs is possible, even if only a few larger sized individuals spawn in Lake IJsselmeer or Markermeer.Since landlocked smelt populations are generally less fecund than estuarine (migrant) smelt populations, a disproportional contribution from estuarine populations could even be expected [22].

The fact that smelt older than two years are rarely observed in Lake IJsselmeer can be explained if they pass the sluices and stay in the Wadden Sea without returning to Lake IJsselmeer. Annually (with a peak in October) large numbers 0 group smelt are passing the sluices to the Wadden Sea (195 tonnes, 65.000.000 individuals, which roughly equals one third of the total IJsselmeer population (Drost & de Witte unpubl.).

^88^Sr levels for the Wadden Sea fish were highly variable. The Wadden Sea is a very dynamic habitat with strong variations in salinity (24–32 PSU [23]) caused by the tide and the outflow of freshwater through the sluices in the Afsluitdijk. Although ^88^Sr values from the Wadden Sea are not available, they are likely to vary greatly and given the strong positive correlation between ^88^Sr and salinity, to follow similar seasonal and daily fluctuations as salinity (http://wwwold.nioz.nl/nioz_nl/ccba2464ba7985d1eb1906b951b1c7f6.php). This high variability in salinity is likely causing the strong fluctuations in ^88^Sr profiles from Wadden Sea individuals and a small overlap in ^88^Sr values between Wadden Sea and Lake IJsselmeer measured in the outer 50 of the otoliths. Because Wadden Sea samples consisted of specimens that must have been 2–4 years old [18], [22], we expected to find dips in the ^88^Sr profile (other than the core area that was probably deposited in their early life in fresh water), potentially indicating a spawning event in freshwater. The absence of such dips could mean that either these individuals did not go to a freshwater area for spawning, did not spawn yet or did not grow (and feed) while being in their spawning areas. It is known that females leave the spawning areas earlier than males, but it is not known whether time spent in rivers differs between the sexes [24]. In all spawning does not take more than 1–4 weeks [21]. The very high concentrations of >3000 ppm (Fig. 3) may indicate the influence of North Sea water inflow of higher salinity.

### Exchange between Freshwater Areas

The two individuals with a dip in their profile later in life (Fig. 4, 20 and 21) suggest that they moved towards water with a lower ^88^Sr profile than Lake IJsselmeer. The ^88^Sr level is similar to that found in smelt in Lake Markermeer. Another possibility is that they swam up the river IJssel, an important spawning location in former days when the lakes were not closed off from the sea yet [19]. There are records of smelt (ca 20 cm) swarming at the River IJssel mouth and migrating upstream for several tens of kilometres dating back to 1840s [19]. A dedicated search for smelt during the spawning period in the River IJssel mouth, and analyses of their otoliths ^88^Sr profiles could clarify if this scenario currently still happens. These two individuals and the Lake IJsselmeer individuals 38 and 40–55 that show core values closer to the range of Markermeer individuals, could be an indication of exchange between Lake IJsselmeer and Lake Markermeer. The only group that has rather low ^88^Sr values (around 1000 ppm) are the ones caught in the east of Lake IJsselmeer (3^rd^ sample in Fig. 4); they cannot be distinguished from Markermeer individuals. The flat ^88^Sr profiles found in Markermeer probably indicates these fish belong to a very local population. ^88^Sr profiles were very similar within and between hauls. Similar profiles within individuals of one haul but different from another haul can be expected if fish stay together in one school for most of their life and encounter similar circumstances as they move around. Alternatively the similar patterns are caused by the ^88^Sr signature of the specific region, which would indicate that smelt do not move around a lot, a finding which shows agreement with a closely related species the rainbow smelt *Osmerus mordax* [25], [26], [27]

### Implications for Management of Landlocked Population

Our results show that under the current management there is no indication of a strong contribution of the Wadden Sea smelt to the populations of both lakes. Currently the possibilities for smelt to re-enter Lake IJsselmeer through the sluices at either Kornwerderzand or Den Oever are limited to periods when sluices are opened. Passage of the discharge sluices towards the Wadden Sea is only possible shortly after opening and towards Lake IJsselmeer shortly before closure. D iadromous smelt tends to aggregate in front of the sluices in March, where in 2009 ca 50 tonnes of adult fish were caught in the commercial smelt fisheries (Drost & de Witte unpubl.). Given the limited entrance possibilities, management to enhance the contribution of diadromous smelt to the Lake IJsselmeer population should concentrate on improving the opportunity to enter by adjusting the discharge regime or increasing the number of entrance points in the spawning period.
